# Modernizing Brain Injury Services in British Columbia: Developing Research Priorities for Children and Youth with Acquired Brain Injury

**DOI:** 10.3390/healthcare14183016

**Published:** 2026-09-15

**Authors:** Scott Ramsay, Jessica A. Harasym, Whitney MacRae, Dana Seidel, Remei Van Raamsdonk, Julia Schmidt, Molly Cairncross, William Paneka, Noah Silverberg, Paul van Donkelaar, Shelina Babul, Roberta Price, Olivia Hale

**Affiliations:** 1 MacIsaac School of Nursing, Faculty of Applied Science, University of British Columbia, Vancouver, BC V6T 1Z1, Canada; 2 Community Brain Injury Program for Children and Youth, Centre for Ability, Vancouver, BC V5R 5H9, Canada; 3 Department of Occupational Science and Occupational Therapy, University of British Columbia, Vancouver, BC V6T 2B5, Canada; 4 Department of Psychology, Simon Fraser University, Burnaby, BC V5A 1S6, Canada; 5 British Columbia Neuropsychiatry Program, Vancouver, BC V6T 1Z2, Canada; 6 Department of Psychology, University of British Columbia, Vancouver, BC V1V 1Z4, Canada; 7 School of Health & Exercise Sciences, University of British Columbia Okanagan, Kelowna, BC V1V 1V7, Canada; 8 Department of Pediatrics, University of British Columbia, Vancouver, BC V6H 3V4, Canada

**Keywords:** acquired brain injury, traumatic brain injury, pediatrics, research priorities, priority setting, nominal group technique, patient and public involvement, health services research, British Columbia

## Abstract

**Highlights:**

- **What is already known on this topic—**Establishing research priorities through collaborative brainstorming can support the research process from funding through to knowledge mobilization.
- **What this study adds—**This study brainstormed ideas presently need for pediatric ABI research to direct future studies, funding calls, and policy.
- **How this study might affect research, practice, or policy—The top 11 priorities** identify areas in which researchers, research networks, and governments can focus their efforts to improve outcomes for children and youth living with ABI.

**Abstract:**

**Background/Objectives**: Acquired brain injury (ABI) affects tens of thousands of British Columbians, yet research priorities specific to pediatric populations have received limited attention. This transdisciplinary engagement study aimed to identify and rank key research priorities for health and care services for children and youth with ABI in British Columbia, Canada, by bringing together researchers, clinicians, community organization representatives, and people with lived experience (PWLE). **Methods**: A hybrid Nominal Group Technique (hNGT) combining virtual and in-person participation was employed from January to May 2025. Thirty advisory panel members—10 researchers, 13 clinicians, five community organization representatives, and two community members, representing a range of disciplines and geographic locations—participated across three phases: silent idea generation using an online platform, a hybrid round-robin clarification meeting, and priority ranking via an online survey. The hybrid approach was designed to maximize engagement and accessibility while preserving equitable opportunities to contribute. **Results**: The advisory panel contributed 49 initial ideas through the online brainstorming platform. Following round-robin clarification, 28 ideas remained in scope and were consolidated into 19 potential research priorities. Twenty advisory members completed the anonymous final ranking, which identified the 11 highest-ranked research priorities. The highest-ranked priority called for greater diversity among research participants to better represent the varied needs of children and youth with ABI. Seven of the 11 priorities addressed intersecting identities and the inclusion of underrepresented populations. **Conclusions:** This hNGT study identified 11 research priorities that can guide future ABI research. The findings emphasize the need for research that reflects the heterogeneous nature of pediatric ABI across diverse communities and contexts.

## 1. Introduction

Acquired brain injury (ABI)—encompassing both traumatic and non-traumatic injuries—remains a major driver of long-term disability and mortality. Recent findings from the Global Burden of Disease (GBD) Study 2021 demonstrate that global age-standardized traumatic brain injury (TBI) rates among young cohorts have generally declined. In contrast, high-income regions, including North America, have experienced an increase in age-standardized prevalence and years lived with disability (YLDs) [1]. The epidemiology of ABI in British Columbia (BC) reflects these high-income North American trends, characterized by a substantial burden driven by road traffic injuries, falls, and sports-related concussions [2]. However, BC’s epidemiological landscape is also shaped by distinct regional drivers—most notably an ongoing unregulated toxic drug crisis that has led to a sharp rise in non-traumatic toxic-hypoxic brain injuries [3], alongside pronounced health disparities among unhoused, rural, and Indigenous populations [4]. ABI can affect a wide range of outcomes, including physical, cognitive, mental, and emotional health. By 2031, brain injury is projected to be among the most common—if not the most common—neurological conditions affecting Canadians [5].

Establishing research and care priorities in collaboration with key partners and knowledge users is essential because it directs research effort toward the areas most relevant to those users. Such collaborative priority-setting helps ensure that research addresses the most pressing real-world needs rather than solely researcher-driven interests, reduces research waste by focusing on questions that matter to those affected, and increases the likelihood that findings will be implemented in clinical practice [6]. Amid calls for improved representation in ABI research [7], a growing number of ABI studies have involved people with lived experience (PWLE-patients or families), ABI healthcare professionals, and community organization representatives (i.e., non-healthcare) in generating and ranking clinical care and research priorities. For example, priority-setting projects with a clinical focus have identified and ranked treatment outcomes for those with aphasia and their family members [8], post-stroke occupational therapy assessment practices [9], vision screening for service members with TBI [10], intervention activities to support functional communication after TBI [11], and return-to-work outcomes for patients with traumatic injuries [12]. Similarly, studies that identify and rank ABI research priorities have focused primarily on adults, addressing topics such as the intersections between brain injury, mental health, and addiction [13], and priorities for concussion diagnosis, prognosis, and rehabilitation from the perspective of healthcare providers [14].

Few ABI priority-setting studies have focused specifically on research priorities for pediatric populations. Osmond and colleagues engaged patients, caregivers, and clinicians to identify the top 10 concussion research questions for patients of all ages [15]. Participants in that study prioritized research on optimal methods for predicting persisting symptoms after concussion and on determining the appropriate timing of return to activity. By contrast, members of the Society for Academic Emergency Medicine identified optimizing cerebral perfusion as the foremost TBI research priority for pediatric emergency medical services providers and partners [16]. A James Lind Alliance priority-setting partnership for childhood neurodisability likewise included ABI alongside other conditions [17].

The unique needs of children and youth remain a gap in the ABI literature, which has focused largely on improving diagnosis, treatment, and rehabilitation [15]. Despite the substantial personal and societal burden of pediatric ABI, more research is needed on the specific needs of children with brain injuries and their families. Several factors contribute to the nuance and complexity of pediatric ABI. First, the pediatric evidence base is small relative to that of adults, even though ABI is among the leading causes of pediatric trauma and disability [18]. Second, ABI can affect a child and their family in numerous and far-reaching ways. Intersecting factors such as race and ethnicity, languages spoken, family income and resources, and proximity to healthcare services all influence experiences and outcomes related to pediatric ABI [19]. Finally, pediatric ABI research spans a wide range of developmental stages, including infants, children, youth, and the transition to young adulthood. Importantly, pediatric populations have distinct biological, psychological, and social needs that limit comparisons with findings from adult-only ABI research.

Given rising ABI rates and the lack of formal research priorities specific to pediatric populations, establishing current research priorities is an important first step toward addressing the challenges present in the systems that provide ABI care for children and youth (aged 0–24 years). This transdisciplinary engagement exercise aimed to address this gap by bringing together individuals who encounter brain injury in acute care, rehabilitation, and community settings—including researchers, clinicians, community organization representatives, and PWLE—to identify and rank key research priorities for health and care services for pediatrics and those transitioning to adult services in British Columbia, Canada.

## 2. Materials and Methods

**Figure 1:**
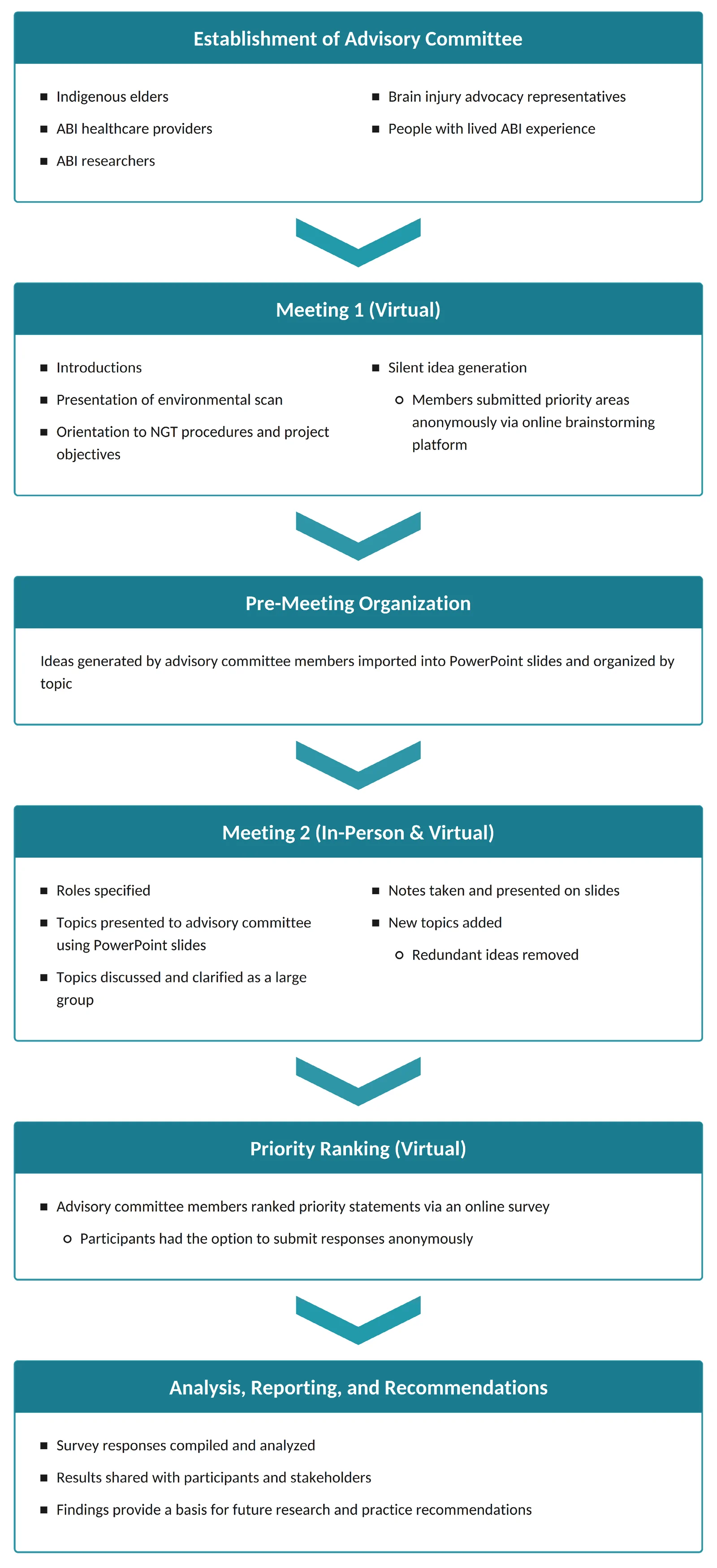
Hybrid nominal group technique procedures.

The Nominal Group Technique (NGT) was used to identify and rank the top research priorities relevant to pediatric ABI in British Columbia, Canada. NGT provides a clear, structured procedure for group decision-making [20] that creates equitable opportunities to contribute and prevents any one individual from dominating the discussion or the resulting consensus [20]. NGT is commonly used in healthcare research to create clinical guidelines [20], identify gaps in clinical services [21], and establish research priorities [22]. The NGT procedure (Figure 1) comprises an introduction to the topic or research question(s), silent idea generation with contributions from all participants, a “round-robin” discussion of the ideas generated that allows participants to clarify the ideas presented, and a final phase of ranking the generated statements or questions [21]. Although researchers initially developed NGT as a face-to-face brainstorming technique, they have since adapted the procedures to include virtual participation [21,22]. Virtual NGT approaches offer increased convenience, reduce geographical barriers, and create opportunities for global collaboration at minimal cost [23]. In contrast, face-to-face participation allows panel members to develop rapport, read and respond to nonverbal communication, and exchange ideas without technical disruption [24]. To capture the advantages of both formats, we developed a hybrid NGT (hNGT) approach in which Meeting 1 was held virtually, and Meeting 2 was held in person with an option to attend virtually. A facilitator experienced in group brainstorming methods led all sessions. The project followed the Reporting Guideline for Priority Setting of Health Research [25]. The project was conducted from 5 January 2025 to 15 May 2025. The research question and the hNGT methodology were developed in collaboration with our community partner to ensure that the study design would meaningfully capture diverse perspectives and create equitable opportunities for participation.

### 2.1. Patient and Public Involvement

People with lived experience of pediatric ABI and their family members were invited to take part in all stages of this research priority-setting study. Given the low response rate to these invitations and recognizing the burden ABI places on families, we invited five representatives from local, provincial, and national brain injury organizations to serve on the 30-person advisory panel alongside researchers and clinicians, as these organizations play an important role in interacting with and supporting PWLE. All committee members had the opportunity to participate in each of the three phases: silent idea generation, in which research priorities were submitted through the online brainstorming platform; the hybrid round-robin clarification meeting; and the anonymous priority-ranking survey.

Their input directly shaped the identification and ranking of the top research priorities. The study employed accessible hybrid methods specifically to reduce barriers and maximize engagement across BC, which is a uniquely large and diverse geographic region in Canada. PWLE and community members are acknowledged in the Acknowledgments section. Because the brainstorming method was anonymous, we cannot identify the stages at which individual members contributed, but we are grateful for their time and input.

### 2.2. Advisory Committee

This exercise aimed to engage participants diverse in expertise, profession, role in ABI services, and years of experience, and involved in or affected by pediatric ABI research. Guided by the United Nations definition for youth [26], the study focused on priorities relevant to children with ABI aged 0 to 24 years old. In NGT, it is recommended to overrecruit before convening the group for brainstorming [27]. As recommended by Smith and colleagues, we employed purposive recruitment based on the lead author and community partners’ relationships. Individuals residing in British Columbia were invited via email to serve as members of the advisory group. Thirty of thirty-three people agreed to participate; this target panel size was based on the investigators’ and community partners’ experience with previous priority-setting work. The panel comprised 10 researchers, 13 clinicians, five brain injury organization representatives, and two community members (Table 1). Community members were those training for health professional roles. This number is over the recommended 8–10 individuals for an NGT exercise [27]. Advisory panel members represented a range of disciplines, including nursing, social work, medicine (with specialties in pediatrics, physiatry, and neurology), neuropsychology, speech-language pathology, occupational therapy, vocational rehabilitation, and education/physical education. In addition to their formal roles, some members self-identified as having lived experience of ABI, either personally as a child or as a family member. Advisory panel members brought expertise in critical care, neurosurgery, concussion care, TBI, epilepsy, sports medicine, oncology, neuropsychology, intimate partner violence, acute rehabilitation, Indigenous health, and outpatient rehabilitation. Given the breadth of the panel’s experience, we decided that a single group was sufficient to conduct the brainstorming exercise. Members were recruited from both rural and urban locations and included multiple Indigenous members. Written informed consent was waived for this quality improvement activity as per the Provincial Health Services sorting tool (#0624) directed by our local institutional review board. All advisory members were informed that participation was voluntary and that an honorarium would be provided for completing the two sessions.

### 2.3. Data Generation

Procedures for the hNGT were adapted from the virtual NGT method described by Riley-Bennett and colleagues [23]. All participants were emailed regular updates about the project, invited to two virtual meetings and one in-person meeting (with the option to attend virtually), and sent links to an online whiteboard for idea generation and an online survey for priority ranking. Categories with small cell sizes (e.g., ethnicity) were collapsed to protect anonymity. Participants reserved the right to withdraw from the session at any point during the ideation or discussion phases without providing a reason. However, due to the anonymous nature of the final voting ballots, individual ranking data could not be selectively retrieved or deleted once it had been submitted into the aggregate tally.

#### 2.3.1. Meeting 1: Introductions and Silent Idea Generation (Virtual)

The first advisory committee meeting, which lasted two hours, was conducted virtually via Zoom. The meeting aimed to introduce the group to one another and provide background information on the research project and the hNGT method for establishing consensus on group-identified research priorities. Twenty-three members attended, and those unable to attend received the agenda and summary notes by email. Silent idea generation was facilitated using the online collaboration tool Padlet [28]; advisory committee members were provided with a link to a private virtual brainstorming board where they could contribute research priorities for pediatric ABI, drawing on their knowledge and experience. Participants could generate as many ideas as they wished, and the brainstorming board provided space to share comments and context for their suggestions. In addition to identifying research priorities, participants also suggested research methods they believed would be useful in exploring these topics. The online idea-generation forum (Padlet) remained active for two weeks after the meeting, allowing committee members to contribute their ideas at their convenience.

#### 2.3.2. Pre-Meeting Organization

At the end of the silent idea generation stage, the research team gathered all advisory member responses and grouped similar statements by topic (e.g., mental health, access to services, equity). Statements were left unedited to preserve participants’ voices. In preparation for the in-person round-robin meeting, responses were entered into PowerPoint slides so the group could view them on a projector screen during the clarification and discussion stage of the hNGT procedures. According to the NGT literature, during this stage of the process, suggestions that appear related can be merged together [27]. Responses were left unedited, although ideas were interpretively grouped (e.g., mental health, access to services, equity) to improve the flow of the in-person discussion.

#### 2.3.3. Meeting 2: Round-Robin Clarification (Hybrid)

The aim of Meeting 2 was to review and clarify all merged brainstormed topics as a group. This four-hour meeting was attended by 19 advisory members. The corresponding author oriented the advisory group to the project’s objectives and clarified team member roles and the procedures for the round-robin clarification stage. An Indigenous Elder opened the meeting with a prayer, provided feedback during the discussion, and closed the meeting with a summary of the day. During the meeting, the 28 slides of collated ideas were projected on a screen and read aloud by the facilitator. Group members were invited to discuss each idea as the facilitator took notes on the slide. Each slide was given 10 min of discussion. The notes remained visible to the group so that participants could build on ideas already generated or clarify any misunderstandings. Virtual participants contributed to the round-robin discussion and viewed the slides and notes through Zoom screen-sharing. As per the NGT literature, if two or more statements appeared comparable, it is useful to merge into a new item. During the discussion, two authors (JH and RVR) compiled the items and drafted a consensus summary statement for each idea (i.e., group agreement by no objections); these statements formed the list of 19 research priorities used in the ranking phase (Table 2).

#### 2.3.4. Priority Ranking (Virtual)

In this phase, the priority statements agreed in Meeting 2 were ranked. All advisory members were invited to review the statements and rank them via Qualtrics, a university-recommended online survey platform on which the data were stored. The survey remained open for two weeks, and members submitted rankings anonymously. The ranking procedure followed established NGT methods [21]. Each member assigned a rank from 1 to 10 to their 10 highest-priority statements from the list of 19 (1 = highest priority, 10 = lowest priority). Ranks were reverse-coded so that a rank of 1 received 10 points and a rank of 10 received 1 point, and the reverse-coded values were summed across members to give each statement a total score, with higher scores indicating higher priority. Independent rankings were aggregated in this way to represent the group’s collective judgment, consistent with previous NGT studies [20,27]. Published NGT studies have used cut-offs of the top 5 [24], 8 [29], or 10 [30] items; we selected the top 10 because 19 statements were carried forward for ranking. The team agreed in advance to retain the 10 highest-scoring priorities and any items tied. A tie at rank 10 resulted in 11 priorities being retained, which are reported here in descending order of total score.

## 3. Results

### 3.1. Gathering Potential Research Priorities

**Figure 2:**
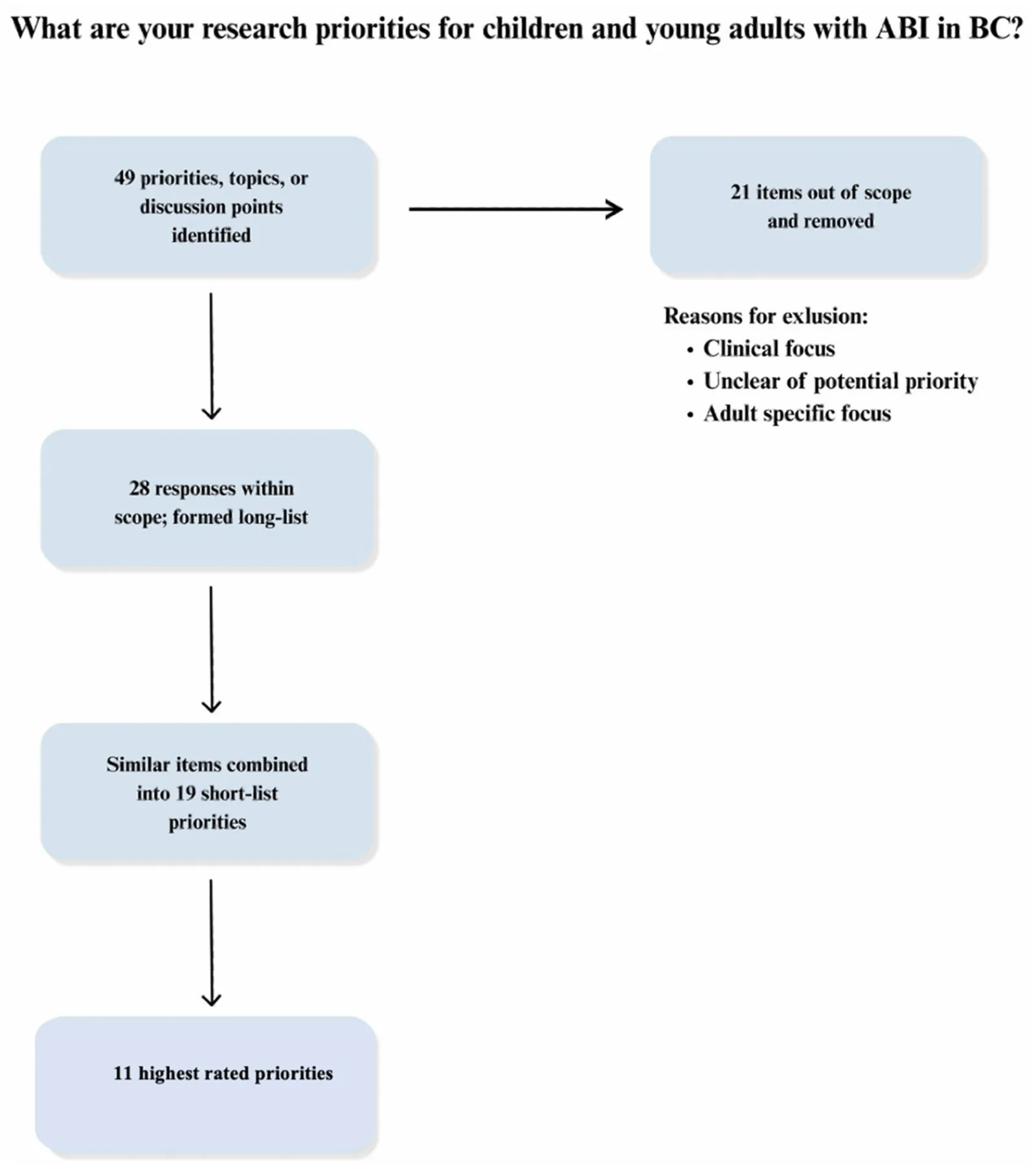
Flow diagram of identification of research priorities.

Advisory members contributed to the Padlet board, creating a shared digital bulletin board of ideas and questions. A total of 49 ABI research priorities (questions and comments–see Supplementary material) were collected from participants during idea generation in Meeting 1 (Figure 2). During Meeting 2, the advisory committee clarified the contributed statements and engaged in a round-robin discussion to determine the scope of the proposed priorities. Of the 49 responses, the advisory committee judged 28 (57%) to be in scope by group consensus and 21 (43%) to be out of scope (e.g., one-word responses, items unrelated to ABI, or items with a purely clinical focus). The 28 in-scope responses were then reviewed by the research team and community partner and merged where they expressed similar ideas at the end of Meeting 2. In total, 19 potential research priorities were identified for ranking (Table 2).

### 3.2. Ranking of Research Priorities

Twenty advisory members fully completed the final priority-ranking survey, with a potential score out of 200 to each statement. The 19 potential research priorities short-listed after Meeting 2 were presented for ranking, and 11 emerged as the final research priorities for ABI research in children and youth in BC (Table 3). Three key areas emerged from the top-ranked priorities. First, participants emphasized equitable representation, prioritizing greater diversity among research participants and the engagement of historically underrepresented groups. Second, they highlighted the importance of incorporating multiple perspectives—particularly lived experience, family, healthcare provider, and community voices—throughout the research process. Third, participants identified critical knowledge gaps requiring attention, including the long-term impacts of ABI on mental health, the need for consistent provincial coding systems to conduct surveillance research, and the relationship between social determinants of health and ABI outcomes.

## 4. Discussion

Using the hNGT as a structured brainstorming procedure, the advisory committee generated 11 research priorities (10 statements, including one tie) for pediatric ABI in BC. The project brought together clinicians, researchers, and community organization representatives from across the province and is, to our knowledge, the first patient-oriented research priority-setting exercise in pediatric ABI. These 11 research priorities identify areas on which researchers, research networks, and governments can focus to improve outcomes for children and youth living with ABI.

The advisory panel ranked most highly those items calling for research that centers the voices of brain injury survivors and their families. One way to achieve this is to involve people with ABI and their families as co-investigators and advisors, from study design through dissemination. Researchers are encouraged to develop research questions in partnership with PWLE and their families and to use study designs that allow participants and knowledge users to co-create knowledge [31]. However, research evidence informed by the lived experience of families is currently lacking in pediatric ABI [32]. Current evidence suggests that systematic planning and multi-partner engagement are needed to successfully translate health research findings into real-world practice improvements [33]. We did include PWLE during the NGT process, although no formal patient or family partners attended meetings. In their place, we had representation from local, provincial, and national brain injury organizations.

The top-ranked research priority identified by our advisory committee was greater diversity among research participants to better represent the diverse needs of children and youth with an ABI (score = 161). Seven priorities, including the top four, relate to the intersecting identities a child or youth may hold. Many factors shape these identities, including socioeconomic status, education level, gender identity, and peer groups. The strong emphasis on diversity and inclusion among the top four priorities (scores ≥ 155) signals evidence gaps that both researchers and knowledge users consider important. Addressing these gaps has the potential to transform how future ABI research is designed and conducted. A review of pediatric concussion research found that Black and Hispanic participants were under-enrolled in epidemiological studies [34]. Similarly, a scoping review of the social determinants of health in pediatric concussion found that race was a frequently reported determinant, and several studies have documented disparities in concussion treatment and outcomes for non-White racialized groups [19]. In the BC context, it is imperative to center Indigenous Peoples’ experiences of brain injury, given the more than 200 First Nations across the province. Lack of diversity in ABI research limits the generalizability of study findings, perpetuates disparities, and contributes to an incomplete understanding of the studied mechanisms [7]. To ensure diverse representation in ABI research, researchers must move beyond convenience sampling at academic centers and actively recruit participants from underrepresented communities, rural areas, and a wider range of injury contexts. Doing so will require researchers and funders to budget for culturally responsive recruitment strategies. For example, research teams can employ community liaisons, reimburse transportation and childcare costs, and develop culturally adapted, back-translated study documents in partnership with community organizations that serve minoritized populations [35,36].

In addition to the need for improved representation and engagement with PWLE in ABI research, one substantive topic emerged: the need for research on the long-term impact of ABI on mental health (priority 5). This priority stands apart from the procedural and methodological areas that dominated the top rankings, suggesting that participants regarded how research is conducted—with diverse, engaged partners—and what is studied as equally critical. The emphasis on long-term mental health outcomes may reflect growing recognition of the persistent psychological sequelae of ABI [37], including depression, anxiety, and post-traumatic stress disorder that can persist years after injury [38]. This priority also aligns with evidence that mental health complications substantially affect rehabilitation outcomes, quality of life, and community reintegration [39], yet remain understudied relative to other sequelae [13].

Improved data collection infrastructure is needed to address the lack of participant representation in current ABI research samples and to study the longer-term impact of pediatric ABI. For example, advanced data systems and consistent brain injury coding platforms (priority 6), are essential infrastructure for the ambitious longitudinal research agendas outlined in priorities 5, 10a, and 10b. Tracking individuals with pediatric ABI across decades requires sophisticated data management capabilities that current systems often lack [40]. Traditional research databases struggle with participant retention, data linkage across healthcare and community settings, and the integration of diverse outcome measures; longitudinal cohort studies face substantial challenges in maintaining contact with mobile populations and tracking families across multiple systems [41]. Without robust, interoperable data infrastructure capable of linking pediatric injury data with adult outcome data across multiple sectors, researchers cannot adequately address questions about how childhood ABI shapes academic trajectories, workforce participation, social integration, or vulnerability to subsequent injuries. Current TBI data systems face significant limitations, including variability in coding practices that produce widely divergent incidence estimates and challenges in capturing the full spectrum of injury severity [42]. The emphasis on developing these systems suggests a recognition that methodological innovation is a prerequisite for answering substantive questions about the long-term impacts of ABI.

### 4.1. Implications for Practice and Policy

The advisory committee’s emphasis on engaging historically underrepresented groups (priority 2) suggests that barriers to care—whether related to transportation, cultural stigma around brain injury, language accessibility, or mistrust of healthcare systems—must be systematically identified and addressed. Socioeconomic disparities, sociocultural influences, and discharge to underprepared communities are major barriers to the accessibility, quality, and efficiency of rehabilitation after ABI [19]. The committee’s focus on including multiple perspectives (patient, family, community, provider) in pediatric ABI research (priority 4), suggests that care models should be expanded to incorporate family expertise and preferences through a family-centered approach, rather than operating on a purely clinician-driven model [42]. Ultimately, these priorities should guide near-term research and, in turn, inform longer-term funding decisions about clinical care that addresses current inequities for people living with ABI.

### 4.2. Limitations and Future Considerations

Several important considerations should be noted when interpreting the findings from this priority-setting exercise. First, advisory members’ backgrounds and experiences inevitably shaped which research areas were identified and prioritized. These research priorities represent the priorities of this specific British Columbia advisory panel. The perspectives, expertise, and lived experiences that individuals brought to the brainstorming and ranking processes directly influenced what was deemed important and worthy of future investigation. Consequently, the research priorities identified in this study reflect the collective viewpoint of this particular panel composition rather than a universal or objective hierarchy of needs. For example, priorities 3 and 4 are closely related statements that may split scores while simultaneously creating the appearance that several independent priorities support the same broad concept. To improve the representation of PWLE, it is recommended that future brainstorming activities ensure that the advisory committee includes an equal proportion of patients and families with diverse backgrounds and experiences. Although 20% of members reported lived experience of brain injury, additional information regarding their specific experiences was not gathered given the nature of initial study design and due to the anonymous nature of the committee member contributions, information about the specific stages they participated in during this brainstorming exercise was not recorded. We acknowledge that our panel was predominantly English-speaking and based in metropolitan areas of BC. Future work should also take additional steps, such as providing translation and interpretation services, to include those who do not speak English as a primary language and include young children and their family members.

Second, the choice of priority-setting methodology carries important implications for study outcomes. This study employed a hybrid NGT, selected specifically for its capacity to manage differences in power and privilege during both the idea-generation and ranking phases. Nonetheless, potential power imbalances remained, including differences among members in their confidence and capacity to contribute during the brainstorming exercise. Although this approach helped mitigate some participatory inequities, future studies should continue to employ methods that democratize the priority-setting process and create space for voices from historically marginalized or underrepresented groups. In addition, although we describe a novel hybrid approach to NGT, attrition still occurred as 20 of the 30 advisory members completed the final ranking.

## 5. Conclusions

This priority-setting exercise brought together researchers, clinicians, brain injury organization representatives, and community members to identify the top priorities for ABI research in BC. Given the high prevalence of ABI, research in this field must not only become a priority but also be reoriented to reflect changing needs of children and youth living with their injuries. These priorities are intended to set objectives for funding calls and to serve as a call to the research community to address these pressing issues.

## Figures and Tables

**Table 1:** Characteristics of Advisory Members.

Characteristic	n (%)
Type of participant	
Researcher	10 (33.3)
Clinician	13 (43.3)
Community organization representative	5 (16.7)
Community member	2 (6.7)
Sex	
Female	20 (66.7)
Male	10 (33.3)
Language spoken	
English	29 (96.7)
Other	1 (3.3)
Geographic location	
Lower Mainland	22 (73.3)
Vancouver Island	4 (13.3)
Okanagan/Interior	3 (10.0)
Other	1 (3.3)
Lived experience	6 (20.0)

**Table 2:** Unranked list of 19 research priorities for children and youth with ABI in BC.

Research Priority
The long-term risks associated with childhood ABI The long-term impact of ABI on mental health The long-term impacts of ABI on academic performance, employment, and social integration in youth The long-term impact of ABI on social functioning The relationship between children with ABI and rates of bullying, discrimination, and social media victimization The relationship between different contexts, services, and outcomes for individuals living with ABI Factors related to sustaining a brain injury through violent mechanisms Current services for pediatric stroke patients in BC Engaging participants from historically underrepresented groups Increasing the diversity of research participants to better represent diverse needs Developing and applying a consistent coding system for patients with ABI in BC to assist with the gathering of epidemiological data Focusing on gathering a variety of outcome data at different levels of care to characterize patient progress Using cohort studies to examine outcomes and impacts of ABI over time Gathering more data on the association between the social determinants of health and ABI Including multiple perspectives, particularly those of the patient, family, and care provider Including people with lived experience of ABI and their family members as members of the research team Employing multiple research methods, including qualitative and quantitative methods (e.g., co-design methods, patient-reported outcomes using surveys and interviews, questionnaires) Adapting data collection methods to improve accessibility for participants with lived experience of ABI Engaging community partners that provide ABI services in all stages of the research process to ensure research activities align with service users’ needs

**Table 3:** Top 10, plus ties, 11 ranked research priorities for children and youth with ABI in BC.

Rank	Research Priority (Ranking Score)
1	Increasing the diversity of research participants to better represent diverse needs (161)
2	Engaging participants from historically underrepresented groups (160)
3	Including people with lived experience of ABI and their family members as members of the research team (156)
4	Including multiple perspectives, particularly those of the patient, family, and care provider (155)
5	Studying the long-term impact of ABI on mental health (154)
6	Developing and applying a consistent coding system for patients with ABI in BC to assist with the gathering of epidemiological data (150)
7	Adapting data collection methods to improve accessibility for participants with lived experience of ABI (148)
8	Gathering more data on the association between the social determinants of health and ABI (147)
9	Engaging community partners that provide ABI services in all stages of the research process to ensure research activities align with service users’ needs (146)
10a	Studying the long-term impacts of ABI on academic performance, employment, and social integration in youth (140)
10b	Studying the long-term risks associated with childhood ABI (140)

## Data Availability

The data presented in this study are available on request from the corresponding author. Requests will be reviewed for merit and conflicts of interest. Please email scott.ramsay@ubc.ca to make your request.

## Supplementary Materials

The following supporting information can be downloaded at: https://www.mdpi.com/article/10.3390/healthcare14183016/s1, Original Contributions for Research on Children and Youth living with ABI in BC.

## Abbreviations

The following abbreviations are used in this manuscript:

ABI	Acquired brain injury
BC	British Columbia
hNGT	Hybrid Nominal Group Technique
NGT	Nominal Group Technique
PWLE	People with lived experience
TBI	Traumatic brain injury

## References

[1] Chen Y., Yang J., Zhang N., Chen Y., Yang J., Zhang N., Tong M., Chen L., Li Y., Dong W. (2026). The global burden of traumatic brain injury in adolescents and young adults, 1990–2021: A systematic analysis for the Global Burden of Disease Study 2021. Ann. Med..

[2] Government of Canada Traumatic Brain Injuries. https://health-infobase.canada.ca/brain-injuries/.

[3] Russolillo A., Ramsay S., Goodyear T. (2025). Anoxic brain injury among people with mental health and substance use concerns: A commentary. Can. J. Addict..

[4] Smye V., Browne A.J., Josewski V., Keith B., Mussell W. (2023). Social Suffering: Indigenous Peoples’ Experiences of Accessing Mental Health and Substance Use Services. Int. J. Environ. Res. Public Health.

[5] Gaskin J., Gomes J., Darshan S., Krewski D. (2017). Burden of neurological conditions in Canada. Neurotoxicology.

[6] Cooke J., Ariss S., Smith C., Read J. (2015). On-going collaborative priority-setting for research activity: A method of capacity building to reduce the research-practice translational gap. Health Res. Policy Syst..

[7] Wiseman-Hakes C., MacDonald S., Keightley M. (2010). Perspectives on evidence-based practice in ABI rehabilitation. “Relevant Research”: Who decides?. NeuroRehabilitation.

[8] Wallace S.J., Worrall L., Rose T., Le Dorze G., Cruice M., Isaksen J., Kong A.P.H., Simmons-Mackie N., Scarinci N., Gauvreau C.A. (2017). Which outcomes are most important to people with aphasia and their families? An international nominal group technique study framed within the ICF. Disabil. Rehabil..

[9] Stapleton T., Connelly D. (2010). Occupational therapy practice in pre-driving assessment post-stroke in the Irish context: Findings from a nominal group technique meeting. Top. Stroke Rehabil..

[10] Radomski M.V., Finkelstein M., Llanos I., Scheiman M., Wagener S.G. (2014). Composition of a vision screen for service members with traumatic brain injury: Consensus using a modified nominal group technique. Am. J. Occup. Ther..

[11] Larkins B.M., Worrall L.E., Hickson L.M. (2004). Stakeholder opinion of functional communication activities following traumatic brain injury. Brain Inj..

[12] Bridger K., Kellezi B., Kendrick D., Kettlewell J., Holmes J., Timmons S., Andrews I., Fallon S., Radford K. (2022). Patients’ views on which return-to-work outcomes should be prioritised: A nominal group technique focus group. Br. J. Occup. Ther..

[13] Kennedy C.J., Woodin E., Schmidt J., Biagioni J.B., Garcia-Barrera M.A. (2024). Ten Priorities for Research Addressing the Intersections of Brain Injury, Mental Health and Addictions: A Stakeholder-Driven Priority-Setting Study. Health Expect..

[14] Smith M.J., Major B.P., Cowen G., Fini N.A., Grant S., Kramer S.F., Hamilton M.J., Lawlor K., Patterson B., Salberg S. (2025). Research priorities for diagnosis, prognosis, and rehabilitation following concussion: Results from a national survey of Australian health professionals. Disabil. Rehabil..

[15] Osmond M.H., Legace E., Gill P.J., Correll R., Cowan K., Dawson J.E., Duncan R., Fox E., Gupta K., Kolstad A.T. (2023). Partnering with Patients, Caregivers, and Clinicians to Determine Research Priorities for Concussion. JAMA Netw. Open.

[16] Adelgais K.M., Hansen M., Lerner E.B., Donofrio J.J., Yadav K., Brown K., Liu Y.T., Denslow P., Denninghoff K., Members of the SAEM Consensus Conference Emergency Medical Services Subcommittee (2018). Establishing the Key Outcomes for Pediatric Emergency Medical Services Research. Acad. Emerg. Med..

[17] James Lind Alliance Childhood Disability. National Institute for Health and Care Research James Lind Alliance Priority Setting Partnerships. https://jla.nihr.ac.uk/priority-setting-partnerships/childhood-disability.

[18] Palanivel V., Burrough M. (2021). Acquired brain injury in children, and their rehabilitation: Where we are now?. Paediatr. Child Health.

[19] Ramsay S., Harasym J.A., Liu B., Lloyd H. (2025). The Social Determinants of Health in Pediatric Concussion: A Scoping Review. J. Head Trauma Rehabil..

[20] Van de Ven A.H., Delbecq A.L. (1975). The effectiveness of nominal, Delphi, and interacting group decision making processes. Acad. Manag. J..

[21] McMillan S.S., King M., Tully M.P. (2016). How to use the nominal group and Delphi techniques. Int. J. Clin. Pharm..

[22] Lee S.H., Cate O.T., Gottlieb M., Horsley T., Shea B., Fournier K., Tran C., Chan T., Wood T.J., Humphrey-Murto S. (2024). The use of virtual nominal groups in healthcare research: An extended scoping review. PLoS ONE.

[23] Riley-Bennett F., Russell L., Fisher R. (2024). An example of the adaptation of the Nominal Group Technique (NGT) to a virtual format (vNGT) within healthcare research. BMC Med. Res. Methodol..

[24] Wheatcroft J., Lannin N.A., Baker A.M., Unsworth C.A. (2025). Expert Consensus on a Cognitive Rehabilitation Learning Package for Novice Occupational Therapists. OTJR Occup. Ther. J. Res..

[25] Tong A., Synnot A., Crowe S., Hill S., Matus A., Scholes-Robertson N., Oliver S., Cowan K., Nasser M., Bhaumik S. (2019). Reporting guideline for priority setting of health research (REPRISE). BMC Med. Res. Methodol..

[26] United Nations Youth. https://www.un.org/en/global-issues/youth.

[27] Smith D., Cartwright M., Dyson J., Aitken L.M. (2023). Use of nominal group technique methods in the virtual setting: A reflective account and recommendations for practice. Aust. Crit. Care.

[28] Rath A. (2025). Padlet: A tool for fostering collaborative learning and feedback literacy in dental education. Front. Med..

[29] Claxton J.D., Ritchie J.B., Zaichkowsky J. (1980). The nominal group technique: Its potential for consumer research. J. Consum. Res..

[30] Hiligsmann M., van Durme C., Geusens P., Dellaert B.G., Dirksen C.D., van der Weijden T., Reginster J.Y., Boonen A. (2013). Nominal group technique to select attributes for discrete choice experiments: An example for drug treatment choice in osteoporosis. Patient Prefer. Adherence.

[31] Hawke L.D., Dada-Phillips W., Seiyad H., Orson J., Goldsmith L., Conway S., Jordan A., Sheikhan N.Y., Hiebert M., Kidd S. (2025). Best Practices Guidelines for the Engagement of People with Lived Experience and Family Members in Mental Health and Substance Use Health Research: A Modified Delphi Consensus Study. Health Expect..

[32] Jenkin T., D’Cruz K., Anderson V., Scheinberg A., Knight S. (2023). Family-centred service in paediatric acquired brain injury rehabilitation: Perspectives of children and adolescents and their families. Disabil. Rehabil..

[33] Tan A., Nagraj S.K., Nasser M., Sharma T., Kuchenmüller T. (2022). What do we know about evidence-informed priority setting processes to set population-level health-research agendas: An overview of reviews. Bull. Natl. Res. Cent..

[34] Wingerson M.J., Smulligan K.L., Svoboda E., Baugh C.M., Magliato S.N., Wilson J.C., Howell D.R. (2025). Who are the participants in pediatric concussion randomized clinical trials? A meta-epidemiology study. JOSPT Open.

[35] Carter C.R., Maki J., Ackermann N., Waters E.A. (2023). Inclusive Recruitment Strategies to Maximize Sociodemographic Diversity among Participants: A St. Louis Case Study. MDM Policy Pract..

[36] Mendez-Luck C.A., Trejo L., Miranda J., Jimenez E., Quiter E.S., Mangione C.M. (2011). Recruitment strategies and costs associated with community-based research in a Mexican-origin population. Gerontologist.

[37] Rees L., Janzen S., McCarthy D., Weiser M., Teasell R., Marshall S., Teasell R., Cullen N., Marshall S., Janzen S., Faltynek P., Bayley M. (2019). Mental health issues post acquired brain injury. Evidence-Based Review of Moderate to Severe Acquired Brain Injury, Version 13.0.

[38] Carmichael J., Hicks A.J., Gould K.R., Ponsford J., Spitz G. (2023). Ten-Year Cohort Study of Emotional Distress Trajectories After Moderate-Severe Traumatic Brain Injury. Arch. Phys. Med. Rehabil..

[39] Murphy F.C., Peers P.V., Blackwell S.E., Holmes E.A., Manly T. (2019). Anticipated and imagined futures: Prospective cognition and depressed mood following brain injury. Br. J. Clin. Psychol..

[40] Oken E., Bastain T.M., Bornkamp N., Breton C.V., Fry R.C., Gold D.R., Hivert M.-F., Howland S., Jackson D.J., Johnson C.C. (2023). When a birth cohort grows up: Challenges and opportunities in longitudinal developmental origins of health and disease (DOHaD) research. J. Dev. Orig. Health Dis..

[41] Eskenazi B., Gladstone E.A., Berkowitz G.S., Drew C.H., Faustman E.M., Holland N.T., Lanphear B., Meisel S.J., Perera F.P., Rauh V.A. (2005). Methodologic and logistic issues in conducting longitudinal birth cohort studies: Lessons learned from the Centers for Children’s Environmental Health and Disease Prevention Research. Environ. Health Perspect..

[42] Eliassen M., Arntzen C., Nikolaisen M., Gramstad A. (2023). Rehabilitation models that support transitions from hospital to home for people with acquired brain injury (ABI): A scoping review. BMC Health Serv. Res..

