# Supplementary material for: Modernizing Brain Injury Services in British Columbia: Developing Research Priorities for Children and Youth with Acquired Brain Injury
Source: Healthcare (Basel). 2026 Sep 15;14(18):3016. doi: 10.3390/healthcare14183016 (PMC13606803; doi:10.3390/healthcare14183016)
Supplement: Supplementary file 1 [file healthcare-14-03016-s001.zip › healthcare-4452729-supplementary.pdf]

49 Original Contributions for Research on Children and Youth living with ABI in BC

Gaps in ABI service and research for children, adolescents and young adults in BC

- **Concussion —** No provincial centre to house pediatric and adult concussions; no long-term follow-up.
- **IPV —** No pediatric studies on violent mechanisms.
- **Family violence —** Challenges with stigma (people may not be willing to disclose cause of injury), comorbid factors, and knowledge amongst care providers of the potential links between family violence / IPV and brain injury.
- **Transitions —** Broadly, no research into the transition from pediatric services to adult services in those with ABI.
- **Pediatric stroke —** There is not an investigator focused on pediatric stroke, although there are clinicians.
- **Time to care and long waits**
- **Underserved / underrecognized populations —** Identification and diagnosis, treatment accessibility, and research participation in historically underrepresented groups.
- **Concussion —** Limited access to clinical practitioners with the ability to provide 1:1 assessment and guidance to patients and family during the early stages of concussion to optimize early management.
- **Lack of a concussion clinic similar to other provinces**
- **Transition beyond 6 months —** Accessing resources and funding for patients that have long-term, possibly permanent therapy needs.
- **Lack of support for family / caregivers**
- **Concussion —** Key challenges for adolescents in final years of high school with goals of transitioning to university — sometimes in different provinces where there are then sudden drops in access to care. Especially true for patients affected during key years of academic and sport performance who do not meet criteria for any additional services.
- **Difficulty for families to navigate through health services —** What is funded, who provides services, and where to fill in the gaps.
- **Comorbidities —** Many youth with ABI experience comorbid mental health difficulties, substance use disorders, and other conditions. Mental health programs are often reluctant to serve clients with ABI as they feel underprepared, while ABI programs may feel treatment of mental health conditions is outside their scope. These clients may go unserved.
- **Lack of services specific to concussion-related mental wellness**
- **Funding / access to rehabilitation support —** Lack of funding for ongoing ABI rehabilitation at the community level.
- **Transitions —** Big gaps at transition points such as school reintegration and adult services. Limited vocational counselling resources at transition points, particularly moving from high school to post-secondary education or employment. Limited resources once CBIP completes for further therapy, especially when

families do not have access to extended benefits. Limited young adult resources if not with VCH, so GF Strong access is limited.

- **Supports for families (parents, siblings)**
- **Mental health services and access to these**
- **Guidelines for interdisciplinary follow-up care —** Guidelines for children with moderate and severe brain injury would be helpful for practitioners. Follow-up required to about age 24; transition post high school is especially challenging.
- **Access —** Lack of access to rehab services in many rural and remote communities.
- **Return to life rather than return to play —** Unpopular opinion, but move the focus of concussion care to return to life instead of return to play for those with multiple concussions.
- **Gaps in services —** Lack of resources in remote communities in terms of therapists as well as supports in school.
- **Family and caregiver knowledge —** Expanding resources for caregivers to access education about brain injury, perhaps more specific information as deficits vary so widely.
- **ABI impact on psychological and psychiatric health —** Little information on the long-term impact of ABI on longer-term mental health. Would inform need for further resources.
- **Research —** What long-term risks are associated with an ABI in childhood.
- **Pediatric stroke —** Epidemiological data and a map of current services.
- **ABI-specific mental health services and programs**
- **Immediate access to care for diagnosis and assessment**
- **Qualitative data collection**
- **Physicians —** Information for family physicians and clinicians about resources, services, community supports, and rehabilitation for children, youth, and young adults with ABI or concussion.
- **Social networks — awareness and education —** Increasing children's, teens', and peers' understanding of the impacts of brain injury may help maintain friendships and decrease isolation after brain injury.
- **Long-term care and support —** Insufficient long-term support structures, including rehabilitation, vocational training, and supportive housing for the transition to adulthood and independent living.
- **Long-term impacts of ABI on academic performance, employment, and social integration in youth**
- **Lack of provincial data collection around the ABI population —** No statistics or demographic information are collected at a provincial health care level (hospital, rehabilitation, community physician) indicating the number of children and youth per year who experience concussion or ABI.
- **Long-term care —** Minimal access to follow-up care at the health care level outside of community physicians.
- **Siloing of supports —** Difficulty with collaborative care at different levels — for example, acute care/hospital vs. community vs. mental health. No comprehensive place to go for ABI supports. Difficulty "accessing back" once at a community level.

- **Respite —** No respite funding or support for caregivers.
- **Peer support —** Lack of peer connections for children and youth with ABI.

Strategies for identifying and measuring impact of ABI

- **Community Integration Questionnaire —** Use the Community Integration Questionnaire on transition to adulthood.
- **Focus groups and increased use of qualitative information gathering**
- **Qualitative measures with patients and caregivers —** Would be very powerful to understand the deficits experienced by patients and families, particularly in under-resourced regions of the province. Perhaps surveys to past CBI patients as a starting point.
- **Later effects on social functioning —** Study whether children with ABI are more vulnerable to bullying, discrimination, and social media victimization.
- **Patient-reported outcomes —** Surveys and interviews that gather self-reported information about symptoms, pain, fatigue, mental health, and satisfaction with life.
- **Data collection —** Collection of information at hospital and primary physician level to allow for follow-up.

Methods for ABI research across populations

- **Diversity of participants —** Efforts to increase representation within research.
- **Inclusion of co-design methods to include lived experience and family perspectives**
- **Multiple perspectives —** Need for multiple perspectives (many types of roles and stakeholders) and multiple methods (qualitative, quantitative).
- **Cohort studies —** Can follow groups of people with ABI over time to examine outcomes like cognitive recovery or changes in social integration, considering factors such as injury severity, age, or socio-economic background.
